# Complete Genome of Aeromonas encheleia Strain SOD01 Isolated from an Urban Freshwater Stream

**DOI:** 10.1128/mra.00673-22

**Published:** 2022-08-18

**Authors:** Jade Arruda, Taylor Brysgel, Elizabeth Capestro, Isabelle Flaherty, Grace Foltz, Abbey Gardner, Michaela Hunt, Jiana Ingrassia, Sean O’Donnell, Gregory Pappas, Emily Peterson, Sarah Ramsaran, Marcelo Rocha, Laura E. Williams

**Affiliations:** a Department of Biology, Providence College, Providence, Rhode Island, USA; University of Southern California

## Abstract

We isolated Aeromonas encheleia strain SOD01 from an urban freshwater stream in Providence, RI. *De novo* assembly of PacBio RSII data followed by polishing with Illumina MiSeq data generated a complete 4,450,115 bp genome with 61.8% GC content. PGAP annotation predicted 3,877 protein-coding genes, 127 tRNA, and 31 rRNA.

## ANNOUNCEMENT

To isolate prey bacteria for studies of predatory *Bdellovibrio* (1), we soaked a sterile swab in water collected from an urban stream in Providence, RI (41.835 N, 71.443 W) then swabbed trypticase soy agar. After incubating plates at 28°C, we picked a colony and conducted three rounds of streaking. To establish freezer stock, we combined 0.5 mL 50% glycerol with 0.5 mL overnight culture grown in trypticase soy broth (TSB) at 28°C.

Using the Wizard Genomic DNA Purification kit (Promega, Madison, WI), we obtained genomic DNA from overnight cultures grown from freezer stock in TSB at 28°C. We extracted DNA from separate cultures for long- and short-read libraries. For long reads, the University of Maryland Institute for Genome Sciences sheared genomic DNA using g-TUBE (Covaris) at 3400 rpm, and then size selected it on a Blue Pippin instrument (Sage Scientific) with an 11,000 bp cutoff. The library was prepared using SMRTbell Template Prep Kit 1.0 (PacBio) and sequenced with P6-C4 chemistry using one SMRT cell on PacBio RS II. For short reads, the University of Rhode Island Genomics and Sequencing Center sheared genomic DNA using a Covaris S220 focused ultrasonicator. The library was prepared using the PrepX DNA Library kit (TaKaRa Bio), visualized on a high-sensitivity BioAnalyzer chip (Agilent), quantified using the KAPA Illumina Quantification kit (Roche), and sequenced on an Illumina MiSeq to obtain 2 × 250 bp paired-end reads.

Unless otherwise noted, default parameters were used for all software. For *de novo* assembly of the PacBio data (138,319 subreads at N50 12,507 bp), we compared Hierarchical Genome Assembly Process v3 (HGAP3) (2) and Canu 2.2 (3). To test HGAP3, we launched an Amazon EC2 instance of SMRT Portal v2.3.0 and set the estimated genome size at 4.5 Mbp, which yielded a 4,468,532 bp contig. After using BLASTN (4) and EMBOSS 6.6.0.0 extractseq (5) to identify and trim overlap between contig ends, the closed contig was 4,447,847 bp. To test Canu, we set the estimated genome size at 4.45 Mbp, which yielded a 4,464,381 bp contig. After using extractseq to trim overlap based on information from Canu, the closed contig was 4,448,166 bp. Comparison using dnadiff (6) identified only 20 SNVs and 2,559 indel bp between HGAP3 and Canu closed contigs, demonstrating good agreement. We proceeded with Canu output and used BLASTN comparisons and extractseq to rotate the closed contig so that the first base corresponded to the *dnaA* start codon.

For polishing, we aligned raw Illumina MiSeq read 1 and read 2 datasets (5,253,695 reads each) separately to the contig using Burrows-Wheeler aligner “mem” (BWA-mem) 0.7.17 (7) with the option to report all possible alignments for each read. Based on samtools (8) analysis, 5,190,922 R1 reads and 5,087,523 R2 reads aligned to at least one location. We used Polypolish v0.5.0 (9) to remove alignments based on insert size and then correct the sequence. Polypolish reported 456.6× coverage, and dnadiff analysis identified correction of one SNV and 1,949 indel bp, yielding a 4,450,113 bp contig. To confirm, we aligned MiSeq R1 and R2 reads to the corrected contig using POLCA within MaSuRCA 4.0.8 (10). POLCA corrected two indel bp, yielding a final genome sequence of 4,450,115 bp and 61.8% GC. Prokaryotic Genome Annotation Pipeline (PGAP) (11) annotation predicted 3,877 protein-coding genes, 127 tRNA, and 31 rRNA. Digital DNA:DNA hybridization analysis with the Type Strain Genome Server (12) classified SOD01 as Aeromonas encheleia.

### Data availability.

*Aeromonas* sp. strain SOD01 genome sequence was deposited in GenBank (accession no. CP099717). PacBio and MiSeq reads were deposited in the SRA (BioProject accession no. PRJNA852219 and SRA accession no. SRX15851419 and SRX15851420, respectively).
